# Acute Indigestion*Read at meeting of the Hood-Summerville Medical Societies, March 3, 1914.

**Published:** 1914-04

**Authors:** Alf Irby

**Affiliations:** Weatherford, Texas


THE
### TEXAS MEDICAL JOURNAL
Established July, 1885

F. E. DANIEL, M. D., - - - - Editor, Publisher and Proprietor

Published Monthly.—Subscription, $1.00 a Year.

VOL. XXIX. AUSTIN, APRIL, 1914. No. 10.

The publisher is not responsible for the views of the contributors.

### Original Articles.

For Texas Medical Journal.

### Acute Indigestion.*

BY ALF IRBY, M. D., WEATHERFORD, TEXAS.

"Washington, D. C., November 21, 1913. John H. Marble, a member of the Interstate Commerce Commission, died here tonight of acute indigestion, by which he was stricken yesterday in Philadelphia."

In taking this newspaper announcement as the subject for a few remarks, I realize that they will be wholly inadequate and of insufficient merit to deserve the name of "a paper." Nevertheless, however poorly written or however sloven the phraseology may be, it is hoped that they may elicit a discussion.

"He died of acute indigestion." It is reasonable to suppose that he had as a complication or sequellae that convenient and ever present trouble, "heart failure."

In the beginning of this, I want to say that there is no such ailment as independent or idiopathic stomach indigestion in spite of Professor Ewald to the contrary. The nearest approach there is to it is the local or gastric disintegration and decomposition of any albuminous substance taken into the stomach as food, commonly known as ptomaine poisoning, or tyrotoxican poison, coming from the ingestion of poisonous milk, cheese or ice cream. But in either of these, the general system is saturated with toxines peculiar to each before the local symptoms are manifest, for digestion has taken place a priori.

The ingesta acted on by the normal fluids of the stomach is digested and assimilated, and, if bearing the elements of poison,

*Read at meeting of the Hood-Summerville Medical Societies, March 3, 1914.

is rendered suitable for the generation of toxines, saturating the blood current, and producing the attendant gastric symptoms—nausea, vomiting and pain. In all such cases digestion has been all too speedy for the good of the patient. Therefore, we cannot truthfully call it *in*digestion.

Now, in the case of Mr. Marble, I would suppose he had (if we exclude from our consideration ptomaine or tyrotoxican poisoning), a *diseased gall bladder;* it may have been only a simple infection of the organ or perhaps had gone so far as the occlusion of some one of the ducts (hepatic, cystic or pancreatic) by a concretion of cholesterin or inspisated bile formed in the gall bladder itself or locally in one of the ducts, thereby preventing the outflow of bile—the normal antiseptic for the intestinal tract. Anything interfering with this regular outflow of the biliary fluid always and inevitably brings on gastric indigestion—the symptoms of which may be simply eruction of gases arising from decomposition of the contained fats or of a more aggravated and pronounced localized pain designated as *colic.*

Whenever we find a faulty or retarded excretion, one of the ducts is occluded and we will find the nearest neighboring organ to be suffering in a degree commensurate with the intensity of the organ or organs primarily affected. Consequently, if we have a gall bladder trouble in an exaggerated and severe form, we have resultant severe gastric indigestion.

Now if, by exclusion, we conclude the patient had not this form of indigestion, his symptoms would point to an inflammatory trouble seated lower in the alimentary canal, giving rise to intestinal indigestion, and we would be likely to find tenderness about McBurney's point with localized or reflex pain—diagnosed as appendicitis.

The last and perhaps the most overlooked and pregnant cause of indigestion is ptoses—the sagging of the various sections of the small and large intestines, especially that of the transverse colon. At the same time the caliber of this sagging bowel is narrowed by congenital bands or from an inflammatory process, and as a result of this mechanical deficiency (often present in the alimentary tract from stomach to sigmoid pouch) there is a lack of the onward flow of the bowel contents, a tardy peristalsis and topical stasis.

When any one of the putrefactive alkaloids is sufficiently fertile with its characteristic toxines and that transitional stage of putrefaction has been reached and the bacteria have begun their invasion of the organic matter, we have as a result a chronic poison and I

believe we can truly call the poison resulting from this very ptoses mentioned above, chronic ptomaine poisoning.

Having referred briefly to the internal causes of ptoses, I desire to mention one of the most potent *external* causes of this condition—the contrivance worn by women to preserve the figure—the corset, with or without its accompanying hose supporters, producing corset stomach, corset liver, corset colon and displacement of many other of the internal organs. Some of the symptoms arisinb from ptoses of these various organs are: First, the displacement of the liver, pains in the hepatic regions, eructations, reflex pains about the shoulders and general feeling of discomfort with attendant hypochondria. Second, from the stomach, local nausea, pain reflexed to the frontal region of the brain, called by the laity sick headache, by the profession migraine. Third, from the colon and lower small intestines, chronic obstipation and constipation, a constant feeling of distention and languor. There is also always present an anemic and sallow cast of the skin.

While this paper is of rather a rambling nature and this is not altogether apropos, still I want to mention it. A few years since a patient was suffering from a chronic ailment that had occasioned him to consult some of the leading men of this and other countries to diagnose his case. Having so far failed, he visited the elder De Costa (the great diagnostician of Philadelphia), who, after thorough and repeated examinations, concluded and so pronounced it partial occlusion of the thoracic duct about four inches above the receptaculum chyli (that trough-like portion of the duct which gathers up the incoming milky fluid known as chyle). The symptoms of this strange and unique trouble were described as follows: A vague, colicy sensation, centrally located about the spine opposite the extreme lower lumbar region, with its reflexes extending laterally up either side to the axillaries, then along the lower border of either scapula, terminating at the inner lower angle of each. The patient was also extremely anemic. He died, and a postmortem verified De Costa's diagnosis.

This leaves me to wonder if this condition is not more often present than we think, and, also, how much it has to do with leukemia or pseudo-leukemia.

I believe that there are a number of undefined troubles with their reflexes of pain and discomfort that are chronically progressing on and on, whose causes ignorance has failed to determine. It yet remains for the profession with its advanced research and strenuous endeavor to discover these causes, apply to them their appropriate names and locate their topography.

I do not want to be understood as trying to write a critique on the nomenclature of diseases, for I am not competent nor equal to the task, but I do think that everyone should bring to bear all known and available means, by exclusion and analysis, to determine the location and cause of familiar diseases and not dispose of them as is so often done as acute indigestion, announcing this the cause of the patient's death.

